# A State of the Art Review on the Use of Natural Fibers in Asphalt Mixtures

**DOI:** 10.3390/ma19040756

**Published:** 2026-02-15

**Authors:** Haichuan Jia, Xin Gao, Yuxin Zhang, Xianghe Meng, Xinyuan Huang, Kun Wang, Long Chen, Peng Hu, Yanping Sheng

**Affiliations:** 1 School of Civil Engineering, Shandong Jiaotong University, Jinan 250357, China; jiahaichuan@sdjtu.edu.cn (H.J.);; 2 Shandong Key Laboratory of Technologies and Systems for Intelligent Construction Equipment, Jinan 250357, China; 3 School of Material Science and Engineering, Chang’an University, Xi’an 710064, China

**Keywords:** natural fiber, fiber asphalt binder, fiber asphalt mixture, road performance

## Abstract

Natural fibers have attracted increasing attention as eco-friendly and sustainable additives for improving the durability and mechanical performance of asphalt mixes. This paper presents a critical state-of-the-art review of the use of six kinds of natural fibers in asphalt mixes. This paper reviews the impact of six natural fibers such as lignin fiber, bamboo fiber, bagasse fiber, corn stalk fiber, basalt fiber, and wool fiber on the properties of bitumen binders and mixes. It examines the influence of these fibers on the physical properties, rheological properties, and fatigue performance of bitumen binders. In addition, the influence of fibers on the moisture stability, anti-cracking, and high- and low-temperature performance of asphalt concrete was analyzed. The review demonstrated that the recommended lengths of natural fibers in asphalt mixes are as follows: lignin fiber 0.8–1.2 mm, bamboo fiber 4–20 mm, sugarcane bagasse fiber 5–12 mm, corn stalk fiber 3 mm, and basalt fiber 6–30 mm. Adding lignin fiber and corn stalk fiber enhanced the high-temperature characteristic of bitumen. The high- and low-temperature properties of the binder were improved by adding bamboo fiber. The addition of basalt fiber and bamboo fiber can increase rutting resistance and fatigue life. Additionally, incorporating the bamboo fiber, bagasse fiber, basalt fiber and wool fiber improved the low-temperature cracking and fatigue resistance of the bitumen mixture. The high-temperature properties of the bitumen mixes were enhanced by using basalt fibers, lignin fibers, bamboo fibers and bagasse fibers. The moisture resistance of bitumen mixes were reinforced by the incorporation of basalt fibers, lignin fibers and bamboo fibers. In general, incorporating natural fibers provided a technical method for improving the performance of asphalt concrete in road applications.

## 1. Introduction

Asphalt pavements have been extensively adopted for road pavement due to the superior functional performance, including excellent skid resistance, noise reduction, riding comfort, rapid traffic opening, ease of maintenance, and recyclability [1,2,3]. However, in recent decades, the rapid growth of traffic volume and the prevalence of heavy loading have posed challenges to the durability of bitumen mixtures [4,5,6]. With the combined impact of mechanical loading and environment, bitumen pavement is susceptible to early deterioration such as cracking, raveling, and permanent deformation which considerably shorten the service life. To address these challenges, extensive research has been directed toward fiber reinforcement [7,8,9]. Numerous research has indicated that fiber incorporation could markedly strengthen the performance of bitumen concrete and improve the lifetime of road pavement [10,11,12].

Currently, the fibers used in asphalt pavements can generally be classified into synthetic and natural fibers. Synthetic fibers mainly include nylon, glass, polyester, steel, aramid, and carbon fibers. Natural fibers mainly include animal, plant and mineral fibers [13,14,15]. Tang et al. [16] researched the interfacial characteristics of bitumen mixtures modified using basalt, glass, and polyester fibers. The results showed that adding fibers improved interfacial bonding strength, with basalt fibers being the most effective. They also found that fiber addition enhanced fracture resistance and the mechanisms of fiber modified bitumen mixtures were studied. Nian et al. [17] examined the impacts of steel fiber regarding the characteristics of bitumen concrete. The findings found that steel fibers incorporation significantly improved resistance to cracking. Serin et al. [18] examined the impacts of fibers on the resistance to cracking of hot mix asphalt (HMA). They found that adding basalt fiber with 0.50% contents provided the greatest fracture energy of bitumen mixes. The basalt fiber with 1.0% content and glass fiber with 0.75% content exhibited the best ductility and fracture toughness indices, respectively. Liu et al. [19] researched the performance of the carbon fiber–graphite hybrid bitumen concrete. The findings demonstrated that the electrical resistivity of the fiber mixture correlated strongly with cracking damage. The study showed that changes in resistivity can provide an early warning of internal failure. The water-damage resistance, rutting resistance and anti-cracking performance could be enhanced. Liu et al. [20] reported that adding bamboo fiber could improve bitumen mixes’ performance. The high-temperature anti-deformation capacity, low-temperature anti-cracking performance, and resistance to moisture damage were reinforced. The findings showed that the dosing of fibers could substantially reinforce the comprehensive performance of bituminous mixes. Fibers of different materials have been reported to optimize interfacial bonding, resistance to low-temperature cracking, high-temperature rutting resistance, moisture resistance, and fatigue behavior. The optimal fiber content and type are crucial, because excessive and insufficient fiber content can reduce the effectiveness. Fiber could also form a reticular structure within the concrete, retarding crack initiation and propagation. Overall, fiber reinforcement was proven to serve as an effective method for reinforcing the durability and mechanical behavior of bituminous mixtures. Fiber addition provided longer service-life and more sustainable pavement materials.

Compared with synthetic fibers, natural fibers exhibited several advantages, such as renewability, low cost, and environmental friendliness. With the increasing focus on green and sustainable development, natural-fiber technology exhibited notable competitiveness for application in pavement engineering [21,22]. However, studies on natural fiber-reinforced bitumen mixture systems remain fragmented. The comprehensive reviews summarizing the performance and practical implications are still lacking. Therefore, a state-of-the-art review is important to provide an in-depth understanding of the potential developments of natural fiber.

The objective of this review is to provide a critical state-of-the-art overview of the use of natural fibers in bitumen and asphalt mixes. The characterization of six kinds of natural fibers was reviewed, such as lignin fiber, bamboo fiber, bagasse fiber, corn stalk fiber, basalt fiber, and wool fiber. The research then studied the performance of these fibers within bitumen. Finally, the influence on the road performance of bitumen mixtures reinforced with fibers was reviewed. This review is intended for researchers and engineers working in the fields of asphalt materials, pavement engineering, and fiber-reinforced composites.

**Figure 1:**
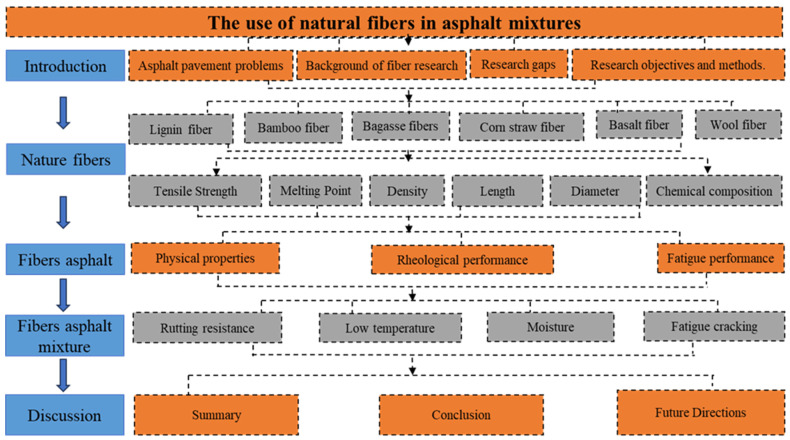
The workflow of the study.

This study adopts a critical review methodology to analyze and synthesize existing research on the application of six kinds of natural fibers in bitumen and bitumen mixes. A literature search was conducted using major scientific databases, including Web of Science, Scopus, and Google Scholar. The journal articles published mainly over the past ten years were focused on. Relevant keywords such as “natural fibers”, “asphalt”, “bitumen”, and “asphalt mixtures” were utilized to identify relevant studies. The selected literature was screened based on the relevance to bitumen materials, clarity of experimental methods, and the road performance results. The studies unrelated to bitumen or lacking sufficient methodological detail were excluded. Key information was extracted from the selected literature, including physical and chemical characteristics of six fibers, and the effects of six natural fibers on the performance of bitumen and bitumen mixes. The physical properties, rheological performance, and fatigue performance of fiber–bitumen and the rutting resistance, low-temperature cracking, moisture, and fatigue resistance of fiber–bitumen mixes were studied. The collected data were then critically evaluated and compared to identify common trends and inconsistencies. Based on the critical analysis, the review synthesized the advantages and limitations of six natural fibers in bitumen applications, and proposed future research directions. The research workflow is shown in Figure 1.

## 2. Characterization of Nature Fibers

### 2.1. Lignin Fiber

**Figure 2:**
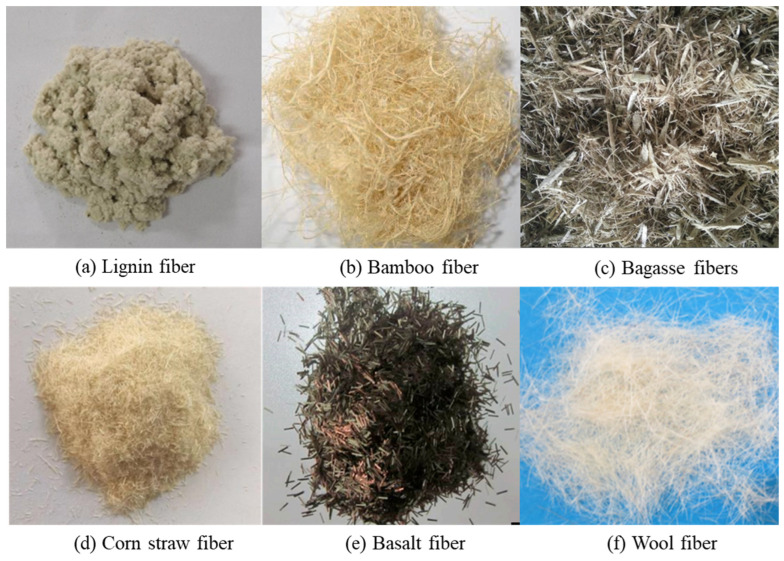
Natural fiber: (**a**) lignin fiber [56]; (**b**) bamboo fiber [57]; (**c**) bagasse fibers [39]; (**d**) corn straw fiber [45]; (**e**) basalt fiber [58]; (**f**) wool fiber [54].

Lignin fiber is a plant-based material obtained from wood through mechanical processing and recycled paper [23,24]. The components of lignin fiber are lignin, cellulose and hemicellulose. The chemical structure is centered on aromatic propane units, which is connected by ether bonds and carbon–carbon bonds. The crystallinity of lignin fiber is relatively low (approximately 30–40%). Lignin fiber exhibits a high specific surface area, and a rough and porous surface texture. The mechanical interlocking, bitumen adsorption, and the stability of bitumen mixes were enhanced. Therefore, lignin fibers are incorporated into bitumen mixes to absorb excess binder, stabilize the bitumen distribution, and enhance mixes homogeneity. These characteristics enable lignin fiber to be regarded as one of the most practical and sustainable natural additives for asphalt pavement engineering [25,26]. As presented in Table 1, the physical characteristics of the lignin fiber utilized in the bitumen concrete were as follows: the tensile strength ranges from 260 to 350 MPa, the density varies between 0.90 and 1.28 g/cm^3^, the fiber length is approximately 0.8–1.2 mm, and the diameter ranges from 8 to 45 µm. The tensile strength begins to decrease at around 200 °C, thus lignin fiber maintains sufficient thermal stability under the mixing and paving temperature of 165 °C. The appearance of lignin fibers is presented in Figure 2a.

Wu et al. [27] investigated the incorporation of anti-rutting agents into lignin fiber-modified mixes. They discovered that the combination of lignin fiber and anti-rutting agents improved properties at high- and low temperatures, moisture stability, and crack resistance. SEM and FTIR analyses showed that lignin fibers formed a network structure and enhanced the long-term performance of bitumen mixes. Zhang et al. [28] investigated the microstructure and mechanical characteristics of fibers in asphalt mixes using resin-impregnated mixture slices and fluorescence microscopy. It was observed that the fibers were predominantly distributed in the internal area of the asphalt mortar and a reinforcing network was formed. Lignin fibers exhibited superior adhesion and mechanical performance compared to polypropylene fibers. The findings demonstrated the lignin-fiber addition was effective in strengthening the micro-mechanical features and performance of bitumen mixes. Pang et al. [29] analyzed the combined use of lignin and ceramic fiber in Stone Mastic Asphalt (SMA) mixes. The findings reported that the addition of lignin fiber significantly improved low-temperature behavior, water resistance, and lifetime. Sheng et al. [30] researched the impacts of different fibers on the volumetric characteristics of SMA mixtures. The study showed that adding lignin fiber slightly influenced bulk specific gravity values within the mineral aggregate and the performance of bitumen mixes was enhanced.

Studies have shown that lignin fiber was used as an effective and sustainable modifier in asphalt mixes. The porous and rough surface of fiber promoted strong interfacial bonding with bitumen and enhanced adhesion with the mixes’ structure. Lignin fibers were capable of absorbing excess bitumen. The binder draindown and bitumen distribution were improved in bitumen mixes, especially in gap-graded concrete. The addition of lignin fiber improved deformation recovery at high-temperature and low-temperature cracking of bitumen mixes. Additionally, lignin-fiber incorporation reinforced the stability of bitumen by reducing bitumen drainage and forming a uniform reinforcement network. Owing to the renewable origin, cost-effectiveness, and reliable reinforcing capability, lignin fiber has become the widely utilized plant fiber in bitumen pavement engineering.

### 2.2. Bamboo Fiber

As a kind of natural fiber, bamboo fiber is obtained from bamboo through mechanical or chemical extraction processing. The initial samples were prepared from bamboo processing waste and were only utilized for laboratory-scale modification research. Bamboo fiber is a natural fiber with a high cellulose content. The component of bamboo fiber is cellulose, hemicellulose, lignin, and a small amount of pectin. Bamboo fiber has a moderate crystallinity. The fiber surface is fibrillated and irregular. The fiber exhibits a high capacity to resist tensile damage and has excellent flexibility. It is a prospective reinforcement material for bitumen applications. Bamboo fiber exhibits good thermal stability and strong oil-absorption ability, which improves the distribution and adhesion of the bitumen within mixes. Additionally, the hollow and void-containing structure of bamboo fiber allows it to absorb a certain amount of asphalt and form a stable network, which enhances the cohesiveness and deformation resistance of asphalt mixes. Incorporating bamboo fiber into binder or mixes could effectively increase the rutting resistance and crack resistance of pavements [31]. As an abundant, renewable, and biodegradable resource, bamboo fiber provides environmental and mechanical advantages, which can be used in sustainable and green pavement engineering. As presented in Table 1, the physical characteristic of the bamboo fiber applied in the bitumen mixes are presented as follows: the tensile strength is 619 MPa, the density ranges from 0.943 to 1.36 g/cm^3^, the fiber length is approximately 4–20 mm, and the diameter ranges from 20 to 87 µm. The fibers begin to decompose at around 260 °C, indicating good thermal stability under typical asphalt mixing conditions. The morphology characteristics of the bamboo fiber is presented in Figure 2b.

Liu et al. [20] assessed the influence of bamboo fiber on the attributes of bitumen mastic and mixes. The research showed that adding bamboo fiber-reinforced resistance to rutting, low-temperature cracking and moisture. The strong bond was formed with the asphalt matrix through adsorption and mechanical anchoring. Cui et al. [32] analyzed the impacts of surface treatment on bamboo fibers and the adhesive property to bitumen. The study showed that silane-treated bamboo fibers exhibited improved interfacial properties, enhancing wettability, rheological property, and fatigue behavior of the asphalt modified with fibers. The treated fiber surface is rough, forming a strong bond with the bitumen matrix. The stiffness and fatigue lifetime of the bitumen mixes was increased, and the phase angle was reduced. Xie et al. [33] researched the effect of basalt and bamboo fibers on the properties of bitumen mastics. The findings indicated that adding fibers could strengthen the resistance to rutting and low-temperature cracking resistance. Cao et al. [34] researched the properties of bitumen modified with bamboo fiber. The study demonstrated that incorporating bamboo fiber significantly enhanced the road performance of bitumen mixes compared to lignin fiber. Shahnewaz et al. [35] researched the impacts of bamboo fiber on porous asphalt (PA) mixes. The findings showed that the addition of bamboo fiber remarkably reinforced the physical and mechanical characteristic.

Bamboo fiber incorporation reinforces the internal structure of asphalt mixes, and the high-temperature stability and low-temperature crack resistance could be enhanced. The moisture susceptibility and durability were enhanced by establishing strong interfacial bonds with asphalt binder. Bamboo fiber addition represents a sustainable and technically reliable alternative for improving the mechanical characteristic and longevity of bitumen pavement.

### 2.3. Bagasse Fibers

Bagasse fibers are derived from the fibrous residue of sugarcane stalks after juice extraction. The production of bagasse fiber is based on the resource utilization of waste residues from the sugar industry. The technology was first pioneered in the late 1990s by Brazil and India. Bagasse fiber is prepared from sugarcane bagasse through the processes of crushing and drying. Bagasse fibers are one of the most abundant agricultural byproducts globally. Bagasse fiber is primarily made up of cellulose, hemicellulose and lignin. A small quantity of ash dominated by potassium and calcium oxides are included. Bagasse fibers show a moderate crystallinity. The fiber surface exhibits a porous and rough morphology. The binder–fiber interaction could be enhanced by the fiber incorporation. Adding fibers to asphalt mixtures results in the recycling of waste materials and improves performance. As presented in Table 1, the physical characteristic of the bagasse fiber adopted for asphalt mixture preparation are as follows: the density is about 1.03 g/cm^3^, the length of the fibers spans a range of 3 to 12 mm, and the diameter is approximately 25 µm. The morphology of the bagasse fiber is illustrated in Figure 2c.

Li et al. [36] researched the degradation of bagasse- and lignin fiber asphalt under hygrothermal cycling. The results showed that hygrothermal aging severely degraded the plant and weakened the binder’s creep recovery. However, bagasse-fiber addition provided aging protection. Meng et al. [37] added the sugarcane bagasse fiber along with polyphosphoric acid into SBS asphalt. The findings demonstrated that the integration of sugarcane bagasse fiber markedly enhanced the performance of the bitumen mixture. Yu et al. [38] developed a novel chemical modification method for bagasse fibers to enhance the interaction with SBS-modified bitumen. The findings demonstrated that chemical modification significantly improved the oil-absorption capacity and thermal stability. Li et al. [39] demonstrated that the incorporation of bagasse fiber significantly enhanced the elevated-temperature stability and low-temperature anti-cracking properties of bituminous mixes. Akarsh et al. [40] researched the practicality of applying sugarcane bagasse ash as a sustainable filler in SMA mixes. The findings proved that sugarcane bagasse ash addition improved the performance in engineering applications of SMA mixtures.

Addition bagasse fibers can strengthen the mechanical and durability performance of bitumen mixes. The bagasse fibers can interact effectively with asphalt binders, and the adhesion, high-temperature stability, and crack resistance of bitumen mixes were enhanced. Chemical and composite modification could enhance the compatibility and adsorption capacity of fiber and resistance against environmental factors. Bagasse fibers exhibit strong potential for mitigating aging and degradation effects under hygrothermal cycling. Moreover, the use of bagasse byproducts as fillers in SMA mixtures has shown improvements in rutting resistance and moisture stability.

### 2.4. Corn Straw Fiber

Corn straw fiber is sourced from the stalks and leaves of corn plants after the harvest of corn kernels. The production of corn stalk fiber began in the early 21st century. The corn stalk fiber was produced by crushing and sieving corn stalks. Subsequent the production process of fiber was improved by the chemical and thermal treatments. Corn straw fiber is an abundant agricultural residue with significant potential applications. Corn straw fibers are lightweight, biodegradable, and possess notable mechanical properties [41]. Corn stalk fiber is mainly composed of cellulose, hemicellulose and lignin. A small amount of lignin–carbohydrate complexes and organosilicon are included. Corn stalk fiber has a moderate-to-low crystallinity. The fiber surface is rough and porous. The fibrous structure improves the mechanical interlocking within the bitumen matrix. The tensile strength and resistance to cracking of bitumen mixes could be enhanced by incorporating corn straw fiber. As presented in Table 1, the density of corn straw fiber is approximately 1.04 g/cm^3^. The fiber length is about 3 mm. The diameter ranges from 50 to 425 µm. The decomposition of the fiber occurs at around 240 °C. The morphology is illustrated in Figure 2d.

Chen et al. [42] assessed the properties of corn stalk fiber-reinforced bitumen. The findings demonstrated that adding corn stalk fiber effectively enhanced the properties. The elevated-temperature stability and low-temperature behavior were enhanced. Hu et al. [43] developed a novel approach for utilizing corn stalk as an asphalt modifier. The study demonstrated that the addition of hydrochar notably enhanced the elevated-temperature stability of fiber–bitumen. Chen et al. [44] utilized corn straw to produce bio-asphalt and the effectiveness was demonstrated. The corn straw-based bio-oil showed excellent compatibility with conventional asphalt, thus the low- and high-temperature properties were enhanced. As reported by Tian et al. [45], the influence of flame-retardant treatment on the performance of corn stalk fibers was researched. The application prospects of treated fibers in asphalt modification were explored. The findings demonstrated that the treated corn stalk fiber significantly upgraded the performance of bitumen. The high- and low-temperature characteristic were strengthened.

The corn straw fiber addition could enhance the asphalt binder properties and provide an effective means of waste utilization. Corn stalk fibers can be utilized to reinforce high- and low-temperature characteristics, due to the uniform dispersion and fibrous structure. The integration of corn straw fibers into asphalt binders and mixture offers performance improvements and cost reduction for pavement construction.

### 2.5. Basalt Fiber

Basalt fiber is classified as a natural fiber, which is derived from the melting and extrusion of basalt rocks. Due to the excellent tensile capacity, thermal endurance and durability, basalt fiber could be a reinforcement material for asphalt mixtures [46,47]. The components of basalt fiber are silicon dioxide and aluminum oxide. A small amount of calcium oxide and magnesium oxide are also included. Basalt fibers exhibit a largely amorphous or partially crystalline glassy structure. The fiber surface is smooth and cylindrical. The fiber incorporation has been shown to reinforce elevated-temperature stability and crack resistance. Moreover, a three-dimensional reticular structure was constructed within the bitumen matrix, which could delay crack initiation and propagation. Basalt fibers are non-combustible and resistant to aging, providing advantages over organic fibers in harsh service environments [48]. Basalt fibers are generally regarded as a distinct category of mineral fibers due to the unique chemical composition and physical properties. Basalt fiber could be classified based on the fiber form and chemical composition. As for the fiber form, basalt fibers are classified as continuous fibers and short fibers. Continuous fibers are mainly used for reinforcing composites. Short fibers are commonly incorporated into asphalt mixes and concrete for crack resistance. Based on chemical composition, basalt fibers can be grouped into high-silica, medium-silica, and low-silica type fiber. A higher silica content provides improved thermal stability and mechanical performance. As illustrated in Table 1, the tensile strength ranges from 2000 to 4800 MPa. The density ranges from 2.56 to 2.72 g/cm^3^. The fiber-length ranges approximately 1.5 to 30 mm. The diameter ranges from 7 to 17 µm. The decomposition temperature exceeds 650 °C. The morphological characteristics are illustrated in Figure 2e.

Li et al. [49] reported that the performance of bitumen was significantly influenced by basalt-fiber diameter. The 16 μm basalt-fiber addition enhanced the elevated-temperature performance and anti-cracking of bitumen mixes. Qin et al. [50] demonstrated that adding basalt fiber significantly enhanced the performance of asphalt mastic. Basalt fiber exhibited superior asphalt adsorption and strength characteristics due to the larger contact area. Zhao et al. [51] demonstrated that adding basalt fiber notably enhanced the pavement service performance of bitumen mixes. Adding basalt fiber improved the Marshall Stability by 19.6% and dynamic stability by 25.5%. The low-temperature bending strain was improved by 22.2%. Long et al. [52] demonstrated that basalt-fiber addition remarkably enhanced the resistance to the fracture of hydraulic bitumen concrete. The study identified that the fracture load and toughness were improved noticeably with 6 mm fiber length and 0.4% content. Wu et al. [53] carried out the comparison of basalt fibers and lignin fibers used in bitumen mixes. The findings indicated that the enhancement in high-temperature performance of basalt fiber mixes was higher than mixture with adding lignin fiber.

Numerous research has demonstrated that adding basalt fiber could clearly upgrade the performance of bitumen and mixes. The fine diameter and strong interfacial bonding of fiber could delay crack propagation and improve load-transfer efficiency. In comparison to conventional fiber mixtures, the basalt fiber mixture exhibits superior high-temperature stability, moisture resistance, and fracture toughness.

### 2.6. Wool Fiber

Wool fiber is a natural animal fiber obtained from sheep and goat. Wool fiber exhibits unique crimped structure, high elasticity, and excellent moisture absorption. Wool fiber is a natural protein fiber composed of keratin, which consists of various amino acids. Wool fiber has a semi-crystalline structure. The fiber surface exhibits a scaly morphology. The elasticity of wool fibers can absorb stress. Incorporating wool fibers into bitumen mixes could strengthen cracking properties and reduce thermal shrinkage. Additionally, wool is biodegradable and renewable. As illustrated in Table 1, the tensile strength ranges are from 1350 to 1580 MPa, the density ranges are from 1.13 to 1.28 g/cm^3^, and the diameter ranges are from 19.5 to 75 µm. The morphological characteristic is illustrated in Figure 2f.

Pirmohammad et al. [54] demonstrated that goat wool fibers significantly enhanced the crack-resistant capacity of bitumen mixes under various loading modes. The findings showed that goat wool fibers exhibited superior performance at shorter lengths. Yousif et al. [55] evaluated the properties of recycled wool fiber-modified bituminous binders. The study demonstrated that wool fiber effectively enhanced the high-temperature behavior of bitumen.

Wool fiber has proven effective in improving the mechanical behavior of bitumen. Studies indicated that wool fibers can enhance fracture resistance under various loading conditions. Additionally, adding wool fibers can improve the elevated-temperature stability and anti-rutting performance of bitumen mixes. The research results exhibited that wool fiber could be a natural reinforcement additives to promote the durability of bitumen pavements.

**Table 1:** The performance of natural fibers in the bitumen concrete.

Fiber	Tensile Strength/MPa	Melting Point/°C	Length/mm	Diameter/µm	Reference
Lignin fiber	300–350	200	3	19	[50]
	<300	-	-	-	[29]
	-	>200	1.10	45	[59]
	<300	260	0.8	8	[60]
	260	240	1.2	16–18	[61]
	-	260	-	8	[26]
Bamboo fiber	619	260	6	-	[62]
	-	-	0–20	20–60	[32]
	-	-	12	29	[63]
	-	-	<6	87	[64]
	619	-	4–8	20–60	[65]
	-	-	<6	-	[66]
Bagasse fibers	-	-	5–10	25	[37]
	-	-	12	25	[63]
Corn straw fiber	-	>240	<3	50–425	[42]
Basalt fiber	3000–4800	650	-	7–15	[67]
	4000–4850	1050	6–15	17	[50]
	>2000	1500–1600	15	11–13	[51]
	>2000	1600	6–30	16	[68]
	3000–4800	650	-	7–15	[52]
	>2000	1550	6	14	[60]
		980	6	13	[69]
Wool fiber	1580	-	-	60–75	[54]
	1350	-	-	19.5	[55]

## 3. Characterization Performance of Nature Fibers in Asphalt Binder

### 3.1. Physical Properties

The physical characteristics of bitumen binder are typically evaluated through softening point, ductility, and penetration tests. The indicators reflect the consistency, temperature sensitivity, and deformation capacity of the bitumen. The penetration test measures the depth to which a standard needle penetrates a bitumen specimen under specified load, time, and temperature conditions. A lower penetration index suggests a stiffer material with superior high-temperature stability and reduced low-temperature flexibility. The softening point test identifies the temperature at which bituminous materials shift from a semi-solid to a viscous liquid phase. A higher softening point indicates improved high-temperature stability and anti-rutting properties. The ductility test assesses the capacity to stretch under tensile stress without breaking, reflecting the ductility and resistance to the low-temperature thermal cracking of bitumen [55,70].

**Figure 3:**
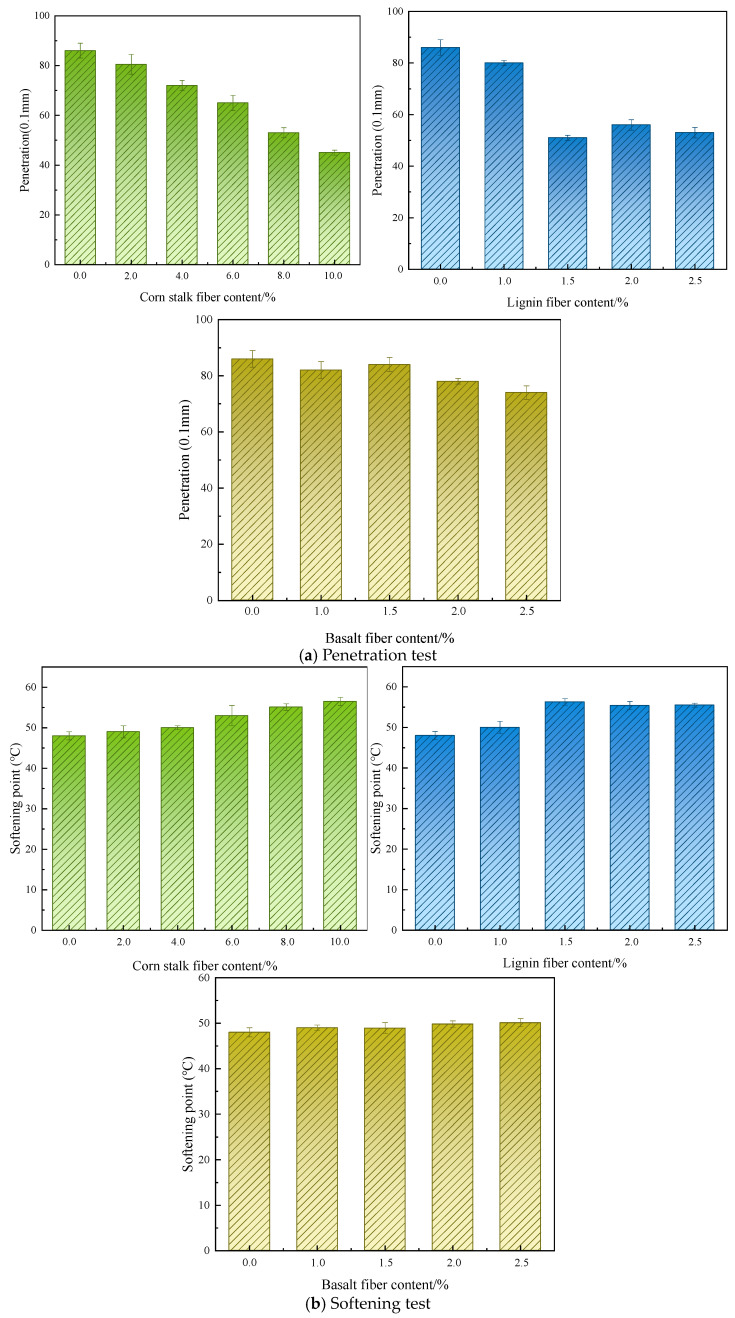
The physical test results of asphalt modified with fiber [42].

Neto et al. [70] researched the properties of lignin-fiber–bitumen using softening point and penetration tests. The findings revealed that incorporating lignin fiber decreased the penetration and increased the softening point of the bitumen. Chen et al. [42] compared the physical and mechanical performance of bitumen incorporated with different fibers using penetration and softening point tests. The findings revealed that the addition of natural fibers generally decreased the penetration and increased the softening point of the bitumen binder, as presented in Figure 3. Tian et al. [45] examined the impacts of corn stalk fiber on the properties of fiber-modified bituminous binders. The findings showed that the penetration of the bituminous binder decreased by 12.8% after fiber addition, indicating that the inclusion of corn stalk fiber enhanced the general viscous properties of the bituminous material. Meanwhile, the softening point of the base asphalt increased by 2.6%, demonstrating improved thermal stability. However, the corn stalk fiber-reinforced asphalt exhibited a 15.9% reduction in ductility. The finding indicated that corn stalk fiber addition could enhance the rigidity and elevated-temperature stability of bituminous binders, but moderately diminish the low-temperature flexibility.

### 3.2. Rheological Performance

Rheological performance describes the viscoelastic characteristics of bitumen across varied temperature levels and loading regimes. It is a critical parameter for studying the anti-rutting and low-temperature cracking characteristics. The Bending Beam Rheometer (BBR) and Dynamic Shear Rheometer (DSR) are the primary experiment for evaluating the rheological properties of bitumen. The DSR test was used to evaluate the elasticity and viscosity components of mechanical response. An elevated G*/sinδ ratio signifies better elastic recovery and anti-rutting performance. A lower G*/sinδ corresponds to improved fatigue performance. The m-value and creep stiffness were assessed by the BBR test, which represents the capability for stress relaxation at low temperatures. A higher m-value and lower creep stiffness indicated superior flexibility and cracking resistance [71,72,73].

**Figure 4:**
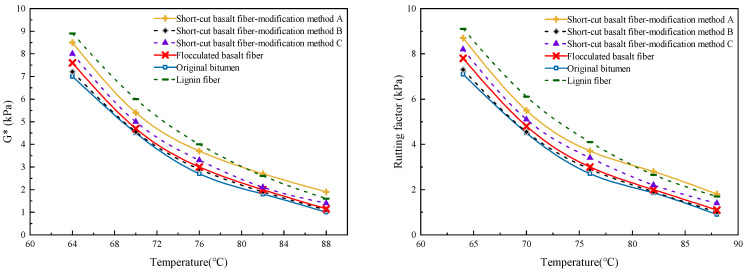
Rheological performance of fiber-reinforced bitumen [53].

Xing et al. [74] researched the impacts of lignin fibers and basalt fiber on the rheological characteristics of bitumen using the DSR and BBR tests. The basalt fibers utilized in the experiment had a length of 6 mm, and the length of lignin fibers was 0.8 mm. The findings showed that the addition of the two fibers enhanced the complex shear modulus and rutting factor. Lignin fiber exhibited a more significant enhancement than basalt fiber, due to the higher surface roughness and stronger asphalt adsorption capacity. However, the incorporation of lignin fiber and basalt fiber negatively affected the low-temperature creep behavior of bitumen. This could be attributed to the increased stiffness and reduced stress relaxation capacity of the bitumen fiber at low temperatures. Wu et al. [53] investigated the impacts of basalt fiber on the rheological behavior of bitumen. Basalt fiber with a fixed length of 6 mm was selected for use in this study. It was concluded that the addition of basalt fibers led to a reduction in the phase angle. The improvement could lead to the reduction in non-recoverable deformation of asphalt under loading. Basalt fiber addition additionally raised the rutting factor and complex modulus, demonstrating improved elevated-temperature stability and anti-rutting properties, as presented in Figure 4. However, the creep stiffness of bitumen was also elevated by the incorporation of basalt fiber, suggesting a decrease in crack resistance and flexibility at low temperatures. Additionally, the research found that the creep rate of the fiber-modified bituminous binder was a superior level compared with that of the unmodified binder at lower temperatures, unlike at higher temperatures. The strong adsorption and interfacial bonding between basalt fibers and bitumen may restrict molecular mobility and hinder viscous flow under low-temperature conditions. Additionally, the basalt fiber-reinforced network may lead to stress concentration and reduced flexibility of the bitumen at low temperatures.

**Figure 5:**
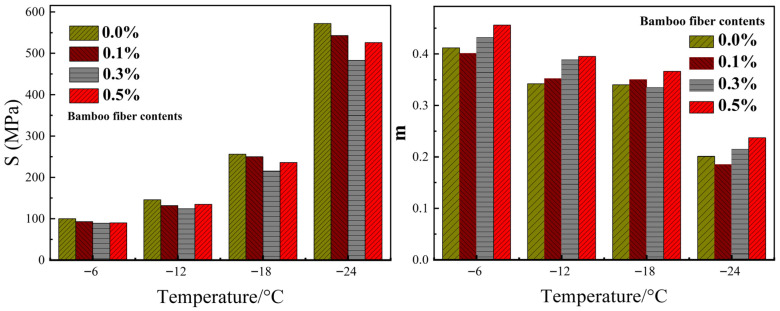
The creep stiffness and creep rate of fiber-reinforced asphalt binders [57].

**Figure 6:**
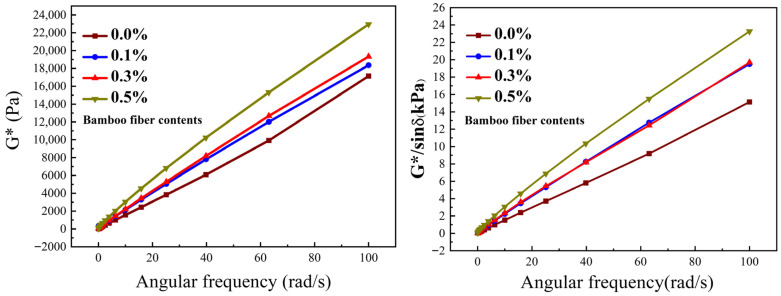
The complex modulus values and rutting resistance factors of fiber-reinforced asphalt [57].

The mechanical behaviors study of bamboo fiber-modified bitumen and the corresponding bituminous mixtures were reported by Jia et al. [57]. The length of bamboo fiber incorporated in the bitumen was 6 mm. The findings revealed the incorporation of bamboo fiber reduced the creep stiffness of bituminous binders, indicating an increase in low-temperature ductility and improved anti-cracking properties. Under the condition of a fixed bamboo fiber content, the creep stiffness exhibited a gradual increase as the test temperature decreased, which confirmed that incorporating bamboo fiber notably improved the bituminous binder’s low-temperature ductility. Under the same temperature conditions, elevating the dosage of bamboo fiber resulted in a steady decrease in creep stiffness, reaching the minimum with the bamboo-fiber dosage controlled at 0.3%. The variation in the m-value with the fiber addition did not show a consistent trend. In some cases, the m-value increased after bamboo-fiber incorporation, while in others it decreased. The fiber addition did not systematically affect the low-temperature stress relaxation characteristics of bituminous binders, as shown in Figure 5. Under elevated temperature (60 °C), incorporating the bamboo fiber notably surged the complex modulus of bituminous binders. Moreover, the rutting factor of bamboo fiber-reinforced bitumen exhibited an upward trend with elevated loading frequency. The rutting characteristic of bitumen mixes was reinforced by incorporating bamboo fiber, as presented in Figure 6.

The increase in the viscosity of bitumen was mainly related to the natural fibers’ absorption of light components of bitumen. The effective free bitumen content was reduced, and the mobility of bitumen molecules was restricted. In addition, fibers with surface roughness could form a spatial network structure within the bitumen. The internal friction and resistance to flow were increased. The elevated-temperature viscosity and stiffness could be reinforced. The enhancement of elastic properties was primarily associated with the mechanical interlocking and stress transfer of the fiber-binder. The elastic recovery of bitumen could be reinforced by the natural fibers, due to bridging micro-deformations and storing elastic energy under loading. Due to the surface adsorption effects, the stiffness of the fiber–bitumen could be improved. Therefore, the rheological improvement of bitumen depends on fiber morphology, surface characteristics, and interaction mechanisms with the bitumen.

### 3.3. Fatigue Performance

The fatigue performance of bitumen refers to the anti-cracking ability of the bituminous binder during long-term lifetime. Wide utilization is made of the Linear Amplitude Sweep (LAS) test to evaluate the fatigue lifetime of the bitumen matrix within the linear viscoelastic domain. From LAS test, the fatigue life, dissipated-energy ratio, and fatigue-damage parameter are derived to quantify the material’s resistance to fatigue cracking [75,76,77].

Li et al. [36] conducted a systematic research into the fatigue performance of natural fiber-reinforced bituminous binders in hygrothermal environments, utilizing the LAS test with reference to the Viscoelastic Continuum Damage (VECD) model. The findings showed that the fatigue life in plant fiber-incorporated bituminous binders was improved compared to unmodified asphalt. However, with the applied strain level and hygrothermal cycling frequency on the rise, the fatigue life of all binders showed a gradual decline. The result suggested that prolonged hygrothermal aging negatively affects the anti-fatigue property of bituminous binders. Cui et al. [32] conducted a study showing the influences of bamboo-fiber incorporation on the fatigue behavior of bitumen. The findings indicated that bamboo fiber-reinforced asphalt exhibited excellent anti-fatigue performance. The silane-treated bamboo fibers notably enhanced the bituminous binder’s fatigue lifetime. The fatigue resistance of bituminous containing treated bamboo fibers exhibited a higher value relative to that of the unmodified asphalt and untreated bamboo-fiber–bitumen. Li et al. [49] researched the impacts of various basalt-fiber varieties on the rheological characteristics of bitumen. The findings showed that at the same strain level, after incorporating basalt fibers of distinct diameter specifications, bitumen exhibited a higher fatigue lifetime compared to the HMA.

## 4. Road Performance of Nature Fibers in Asphalt Mixture

### 4.1. Rutting Resistance

Rutting resistance refers to the capacity of bituminous mixes for inhibiting permanent deformation under sustained repeated traffic loading, especially in high-temperature service scenarios. Rutting typically arises from the accumulation of plastic strain within the bitumen layer, resulting in longitudinal depressions in the wheel paths and severe pavement distress. To examine the rutting characteristic of bituminous concrete, several laboratory tests are widely adopted. The wheel tracking test is the most widely utilized, and the dynamic stability (DS) serves to calculate and quantify the capability of resisting deformation. Additionally, rutting and moisture damage are assessed via the Hamburg wheel-tracking test. Specimens are subjected to repeated wheel loading in a water bath. The repeated load axial test or unconfined dynamic creep test can also be used to measure the flow number and permanent strain, which reflect the mixture’s viscoelastic response under stress [78,79].

**Figure 7:**
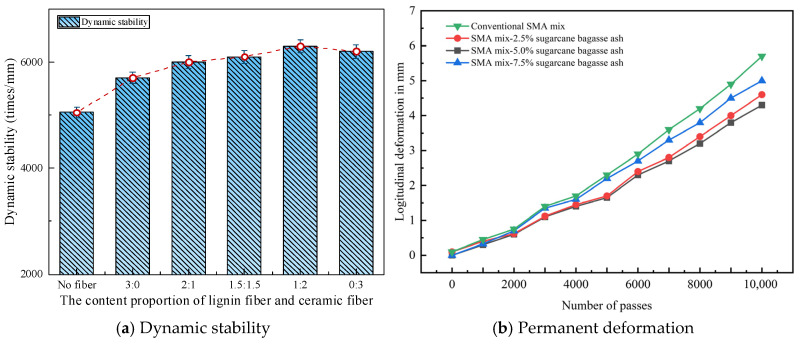
The rutting rest result of fiber-modified asphalt [29,40].

Pang et al. [29] added lignin fibers to bituminous mixes and investigated the rutting characteristics through wheel-tracking tests. The findings showed that the introduction of lignin fiber effectively reinforced the elevated-temperature characteristics of bituminous mixes, as shown in Figure 7a. Ahmed et al. [63] reported the impacts of bamboo fiber on the rutting resistance of bituminous mixtures. The findings revealed that bamboo fibers notably enhanced the tensile mechanical performance of the mixes. Akarsh et al. [40] researched the rutting performance of bituminous mixes reinforced by bagasse fibers. It was found that mixtures containing bagasse fibers exhibited superior rutting-resistance. When bagasse fibers replaced 50% of the ordinary Portland cement filler, the longitudinal rut depth decreased by about 30%, as shown in Figure 7b. Cai et al. [80] investigated the performance of basalt fiber-modified bituminous mixtures through rutting tests. The findings demonstrated that adding basalt fibers observably improved the anti-rutting performance of bituminous mixtures under elevated temperatures.

### 4.2. Low-Temperature Cracking Performance

Low-temperature anti-cracking behavior embodies the capacity of bituminous mixtures to resist tensile stress under low temperatures. The indirect tensile test (IDT) and low-temperature bending test are commonly used to research the properties in asphalt mixes. The bending tensile strength and fracture toughness are commonly used as key evaluation parameters. A higher value indicates better flexibility and crack resistance at low temperatures [81].

**Figure 8:**
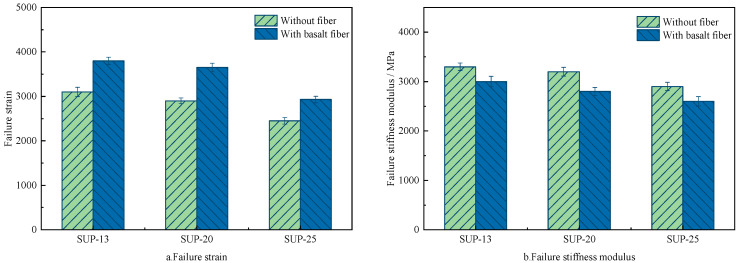
The result of low-temperature cracking performance for fiber-modified asphalt [82].

Abdelsalam et al. [59] incorporated lignin fibers into bituminous mixes and assessed the anti-cracking characteristic at low-temperature. The findings revealed that incorporating lignin fibers elevated the tensile strain and flexural strength of bituminous mixtures. Liu et al. [20] assessed the low-temperature anti-cracking behavior of bituminous mixtures modified with bagasse and bamboo fibers. The findings demonstrated that incorporating plant fibers significantly elevated the low-temperature anti-cracking behavior. The flexural stiffness modulus and peak bending strain of fiber–asphalt mixes were elevated. Li et al. [39] assessed the influence of bagasse fibers on the cracking behavior at low-temperature of bituminous mixes. The results revealed that incorporating bagasse fiber notably improved the anti-cracking capacity at low-temperature. Lou et al. [82] explored the influence of basalt fiber on the low-temperature cracking characteristic of bituminous mixes. The findings showed that fiber addition reinforced the fracture strain and reduced the flexural modulus for different bitumen mixes. Adding basalt fiber could notably enhance the anti-cracking behavior of bituminous mixes at low temperatures, as illustrated in Figure 8. Pirmohammad et al. [54] reported that the fracture strength of bitumen mixes was notably enhanced by wool-fiber addition. The findings indicated that wool fibers showed more significant improvement under pure Mode II scenarios. Additionally, the fiber length and content exerted a pivotal effect on the reinforcing effect. The study found that the optimal enhancement was achieved with 4 mm length and 0.3% content wool fibers.

### 4.3. Moisture Performance

Moisture performance represents the ability of bituminous mixes to resist impairment caused by water infiltration and moisture-induced stripping at the bitumen-aggregate interface. When exposed to repeated wetting-drying or freeze–thaw cycles, moisture penetrates the bitumen mixture. The bonding adhesion between bituminous binders and aggregates was damaged, and the cohesion strength of the mastic was reduced. The process often leads to loss of stiffness, raveling, stripping, and premature pavement failure. The Marshall immersion test and freeze–thaw splitting test (Tensile Strength Ratio, TSR) are broadly adopted. The TSR value from the freeze–thaw test and the residual stability from the Marshall test quantify the retained strength of the mixture after water conditioning. A higher TSR or residual stability indicates stronger water resistance and adhesion between binder and aggregate [83,84].

**Figure 9:**
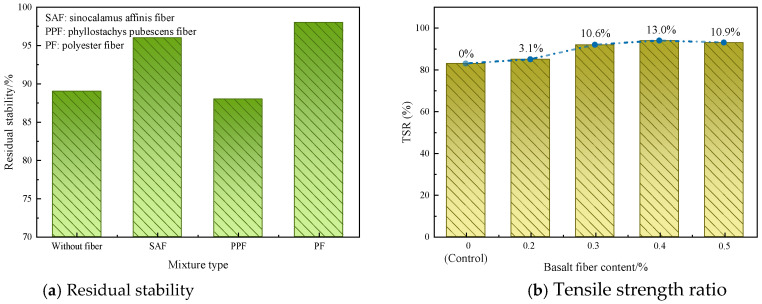
Moisture stability of the fiber–bituminous mixes [86,87].

Wu et al. [85] added lignin fibers to bituminous mixes and evaluated the moisture stability through a freeze–thaw splitting test. The findings showed that the TSR of the lignin fiber-modified mixture increased by 11.4%. Moreover, after freeze–thaw cycles, the tensile strength decreased by only 21.9%. Adding lignin fibers effectively mitigated strength deterioration under moisture conditions. Li et al. [86] researched the influence of bamboo fibers on the water stability of modified bituminous mixes. The findings revealed that incorporating bamboo fiber could strengthen the adhesion of bitumen mixes. The resistance to water-induced damage of bituminous mixture was enhanced, as shown in Figure 9a. Cheng et al. [87] researched the impacts of basalt fibers on the moisture stability of bitumen mixes. The TSR value was notably strengthened with incorporating basalt fibers, as presented in Figure 9b.

### 4.4. Fatigue Cracking Resistance

Fatigue cracking resistance reflects the ability of asphalt mixes were subjected to repeated traffic loading without cracking. Under cyclic stress or strain, asphalt mixes gradually accumulate microscopic damage, which leads to stiffness degradation and crack initiation. The fatigue characteristic of bituminous concrete is commonly assessed via four-point bending tests, fatigue tests, or indirect tensile fatigue tests. The primary assessment parameters are designated as the rate of stiffness modulus decay and fatigue life [88,89,90].

**Figure 10:**
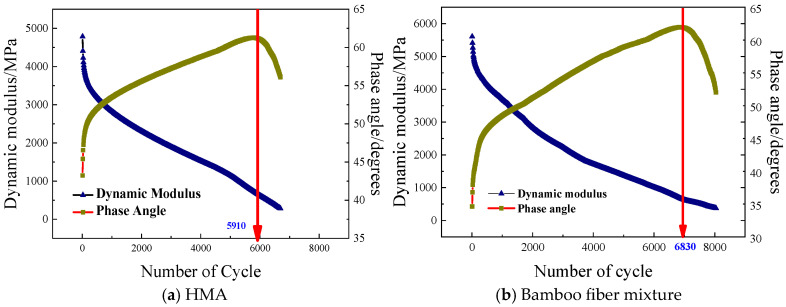
The fatigue lifetime of the bamboo fiber–bituminous mixes [57].

**Figure 11:**
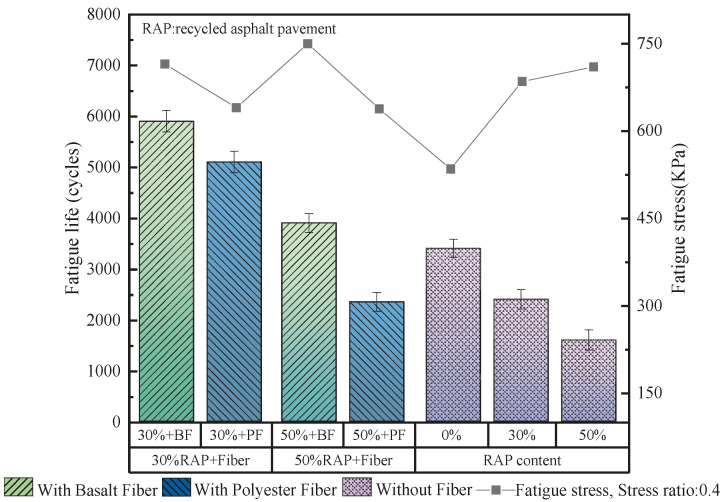
The fatigue test result of the basalt fiber–bituminous mixture [91].

Pang et al. [29] studied the fatigue performance of bituminous mixes modified with lignin fibers. The findings indicated that the incorporation of lignin fiber notably improved the fatigue life and tensile strength. The rutting and anti-cracking capacity of mixtures was enhanced. Jia et al. [57] explored the impacts of bamboo fibers on the anti-cracking performance of bituminous mixes. At 10 Hz and 20 °C, the failure cycles of the HMA and the bamboo fiber-modified mixes were 5910 and 6830, respectively. Incorporating bamboo fibers could effectively reinforce the durability of bituminous mixtures, as shown in Figure 10. Akarsh et al. [40] researched the influence of bagasse fibers on the cracking characteristics of fiber–bituminous mixes. The findings demonstrated that the fiber-modified mixtures exhibited superior fatigue life compared with SMA mixtures under various tensile stress levels. Zhu et al. [91] reported that indirect tensile fatigue tests at a 0.4 stress ratio were used to quantify the influence of basalt fibers on the fatigue anti-cracking capacity of bituminous concrete. When the RAP content was 30% and 50%, adding basalt fibers resulted in a 154% and 135% increase in fatigue lifespan, while polyester fibers improved it by only 119% and 38% under the same conditions. The findings indicated that basalt-fiber addition provided a notable enhancement in fatigue lifetime of bitumen mixes than synthetic fibers, as presented in Figure 11.

As such, incorporating natural fibers significantly enhanced the road performance of bituminous mixes. Lignin-fiber addition reinforced the bituminous-aggregate interface characteristics due to the rough surface and strong adsorption ability. The elevated-temperature stability, anti-cracking and water stability of bituminous mixes was enhanced. Bamboo fibers exhibited superior performance in improving elevated-temperature stability, fatigue durability of bituminous mixes and anti-cracking properties at low-temperature condition, because fiber can bridge microcracks and delay crack propagation under cyclic or thermal stresses. Bagasse fibers provided balanced enhancement across rutting and fatigue performance of bituminous mixes, due to their porous and lightweight nature. In contrast, basalt fibers delivered the most pronounced improvement in elevated-temperature stability, moisture, anti-cracking and fatigue lifespan of bituminous mixes, due to the strong interfacial bonding and network reinforcement effects.

## 5. The Disadvantages of Natural Fibers

Natural fibers showed great potential in improving the properties of bitumen mixes. However, the practical application of natural fiber was influenced by some inherent limitations. The workability of the bitumen mixes could be reduced due to natural fibers tending to absorb bitumen and moisture. The rheological properties of the bitumen and the effective fiber–bitumen interfacial adhesion could be decreased. Additionally, natural fibers could be tangle or clump during the mixing process of bitumen mixes. The effectiveness in enhancing the performance of the bitumen mixes was reduced. The uneven fiber distribution existed in the bitumen mixes. The expected improvement in mechanical properties of bitumen mixes was decreased. Furthermore, some natural fibers were sensitive to the temperatures. Nature fibers were subjected to degradation in an extreme environment, and the tensile strength was reduced. The elevated-temperature performance of the fiber–bitumen mixes was limited and the long-term durability was reduced in an extreme environment. The natural fiber showed poorer thermal stability and susceptibility to environmental factors.

Natural fibers exhibited significant difference in the physical, chemical, and mechanical characteristics due to the origin, species, growth conditions, and processing methods. The different fiber surface, crystallinity and cell-wall structure could lead to inconsistent tensile strength, stiffness, and hydrophilicity of natural fiber. The interactions of the fiber with bitumen was be unpredictable. As such, the difference in reinforcing efficiency of fibers could lead to inconsistent improvements in cracking resistance and durability of bitumen mixes. The variability presented challenges in standardizing fiber specifications and obtaining reproducible performance of fiber–bitumen mixes. The traditional equipment and processes were typically designed for traditional bitumen mixes. The large-scale application of natural fiber-reinforced bitumen mixes was limited due to the lack of experience in handling, mixing, and compaction. The additional equipment calibration, worker training and adjustments to production parameters were required when the existing machinery and established mixing formulas of traditional mixes were incompatible with the fiber–bitumen mixes.

Furthermore, the processing, transportation and storage processes of natural fibers presented numerous challenges. The cost and availability of natural fibers could be fluctuated due to regional agricultural production, seasonality, and processing methods. The practical application in large-scale paving projects was limited. The quality control was significant during fiber production and storage, because the improper handling could lead to fiber degradation, moisture absorption and contamination. In order to prevent the moisture absorption, mold growth, and fiber degradation, the harvesting, drying, and storage of natural fibers need strict environmental conditions. The moisture absorption could cause fibers to swell and decrease in mechanical properties in the bitumen matrix. In addition, when natural fibers were exposed to high humidity and prolonged storage conditions, the fibers were susceptible to microbial attack. The biological degradations and environmental damage could alter the fiber microstructure and reduce tensile strength. The bonding efficiency between the fiber and bitumen was weakened. The reinforcing effect of natural fibers in bitumen mixes was diminished. The specific processing and storage protocols need to be developed to ensure that the fibers maintain the integrity and performance. The review indicated the significance of considering the dispersion, ecological benefits, and the technical and storage challenges during the use of natural fiber in bitumen mixes.

## 6. Further Research Directions

Future research of natural fiber-reinforced bitumen mixes should focus on the six aspects. The interaction mechanisms between the fiber and bitumen mixes should be investigated in-depth. Although existing studies have demonstrated that the natural fibers could reinforced the performance of bitumen mixes, the interfacial bonding, stress transfer, and crack-bridging behavior remain insufficiently studied. Future studies should adopt multi-scale approaches to study the mechanisms between the fiber and bitumen mixes with the integrate microstructural characterization techniques and numerical modeling.

Future research should research the performance evolution of natural fiber-reinforced bitumen mixes throughout the lifetime at the terrible environmental conditioning. It is crucial for evaluating the durability of natural fiber–bitumen mixes and ensuring the long-term effectiveness of fiber in road applications.

Future research should further investigate the synergistic effects of natural fibers in bitumen mixes. The potential effects of combining multiple natural fibers in bitumen mixes could be explored. Different natural fibers vary in geometric shape, stiffness, surface characteristics, and chemical composition. The improvement effectiveness of improving bitumen mixes performance could be enhanced by blending different fibers.

Research on pavement construction-related issues and practical applications of natural fibers should be strengthened. The incorporation of natural fibers could affect the workability, mixing uniformity, compaction performance, and overall construction performance of bitumen mixes. The fiber dispersion efficiency, mixing processes, dosage control, and compatibility with mixing equipment should be studied in the future research.

The environmental and economic benefit assessments should be strengthened. The overall sustainability of natural fiber depends on the production processes, transportation, durability, and life-cycle performance. The life-cycle assessments and cost–benefit analyses should be conducted to balance environmental benefits, performance improvements and construction costs. The assessments could provide quantitative guidance for pavement designers or practitioners.

The standardization of natural fibers utilized in bitumen mixes should be developed. Because the difference in the fiber origin, species, growth environment, and processing techniques, natural fibers could exhibit significant differences in physical characteristic, chemical composition, and mechanical properties. Therefore, the study of unified classification systems and standardized natural-fiber specifications is one goal of the future research.

## 7. Conclusions

This paper characterizes various natural fibers used in the asphalt mixes and pavements. The tensile strength, thermal decomposition temperature, density, length, and diameter of fiber were summarized. The influence of the fibers on the physical and rheological characteristics of bitumen was reviewed. Furthermore, the paper reviewed the impacts of natural fibers on the road properties of bituminous mixes. The conclusions are summarized below:

- Natural fibers was commonly utilized in bituminous mixtures include lignin fiber, bamboo fiber, bagasse fiber, corn stalk fiber, basalt fiber, and wool fiber. The recommended lengths for these fibers in asphalt mixes were 0.8–1.2 mm for lignin fiber, 4–20 mm for bamboo fiber, 5–12 mm for bagasse fiber, 3 mm for corn stalk fiber, and 6–30 mm for basalt fiber.
- Lignin-fiber incorporation reinforced the elevated-temperature stability of bitumen. Due to the lignin fiber with low-crystallinity and a rough surface, bitumen adsorption and the stability of bitumen were reinforced. Lignin-fiber incorporation was more effective at lower doses but agglomerated at higher amounts. The agglomeration led to the decrease in bitumen performance. Corn stalk fibers were characterized by a moderate cellulose content and a rough surface morphology. Corn stalk fiber incorporation increased the softening point and decreased penetration of bitumen. The elevated-temperature performance of bitumen was reinforced. However, incorporating corn stalk fiber could reduce ductility of bitumen and flexibility, because the interaction between fiber and bitumen resulted in reduced flexibility.
- The stiffness and rutting resistance was reinforced with incorporating lignin fibers. This could be attributed to the rough surface morphology and relatively low crystallinity. The effective bitumen adsorption and mechanical interlocking were reinforced. Incorporating basalt fiber notably reinforced elevated-temperature stability due to the high tensile strength and rigid glassy structure. However, the stress relaxation at low temperatures was limited by the smooth surface and high stiffness of fiber. The low-temperature flexibility of bitumen was decreased. The elevated-temperature stability and low-temperature flexibility were reinforced by incorporating bamboo fiber. The performance enhancement was associated with the moderate crystallinity, fibrillated surface morphology, and flexible lignocellulosic structure. Incorporating bamboo and basalt fibers was effective in enhancing the fatigue behavior of bitumen. Because the fibers incorporation could enhance the stress transfer and crack-bridging mechanisms.
- The flexural strength and anti-cracking properties of bitumen mixes were reinforced with incorporating lignin fibers. This is because the fiber–bitumen interlocking and structural fiber-asphalt were formed. The cracking performance at low-temperature was reinforced by incorporating bamboo fiber, basalt fiber and bagasse fiber. Adding bamboo and bagasse fibers with the lignocellulosic structure and moderate crystallinity reinforced the flexibility and crack-bridging capacity of bitumen mixes at low temperatures. Basalt fibers could reinforce the tensile reinforcement due to the high strength. The elevated-temperature rutting resistance could be reinforced by incorporating basalt fibers, lignin fibers, bamboo fibers and bagasse fibers. Because the mixes’ stiffness and load-bearing capacity at elevated temperatures were reinforced. And the wool fibers showed significant increase in fracture strength of bitumen mixes. This is could be attributed to the keratin-based semi-crystalline structure and scaly surface morphology. The mechanical interlocking could be reinforced and the energy dissipation could be reduced during crack propagation.
- The incorporation of lignin fiber and bamboo fiber could reinforce the resistance to freeze–thaw cycles by reducing strength deterioration of bitumen mixes. This is attributed to the fiber–binder interaction was enhanced and the moisture was limited by fiber. Basalt-fiber incorporation also increased the tensile strength ratios and reinforced the moisture stability of bitumen mixes. The fiber exhibited high tensile strength and stable interfacial bonding with the bitumen matrix. Additionally, incorporating the bamboo fiber and bagasse fiber showed substantial improvements in fatigue resistance of bitumen mixes. Basalt-fiber incorporation exhibited the most pronounced enhancement in fatigue lifetime. This is could be attributed to the high strength and strong load-transfer capability at the fiber–bitumen interface.
- The incorporation of natural fibers in the bitumen mixes presents some limitations. Natural fibers could absorb bitumen and moisture, which could reduce the interfacial adhesion between the fibers and bitumen. Furthermore, entanglement and agglomeration of fibers could create weak areas within the bitumen mixes during the mixing process. Natural fibers are also sensitive to the extreme environmental conditions encountered during mixing, construction, production, transportation, and storage. The fiber degradation, reduced tensile strength, decreased stiffness would be existed. Limited construction experience, incompatibility with equipment, and the need for additional process adjustments further hinder large-scale application of natural fiber in the pavement.
- Future research on natural fiber-reinforced bitumen mixes should focus on several aspects. The interaction mechanisms between natural fibers and bitumen mixes could be investigated through multi-scale approaches. Future research should consider the synergistic effects of combining various natural fibers in the bitumen mixes. The on-site construction techniques for fiber–bitumen pavements should be improved to reduce fiber agglomeration. The long-term durability and environmental resistance of natural fiber-reinforced bitumen mixes need be further investigated. The life-cycle assessment and cost–benefit analysis of natural fiber–bitumen mixes need be evaluated in the future. The standardization of natural fibers utilized in bitumen mixes should be developed to reinforce performance reproducibility.

## Data Availability

No new data were created or analyzed in this study. Data sharing is not applicable to this article.
